# Supplementary material for: Co-Formulation of Amphiphilic Cationic and Anionic Cyclodextrins Forming Nanoparticles for siRNA Delivery in the Treatment of Acute Myeloid Leukaemia
Source: Int J Mol Sci. 2022 Aug 29;23(17):9791. doi: 10.3390/ijms23179791 (PMC9456197; doi:10.3390/ijms23179791)
Supplement: Supplementary file 1 [file ijms-23-09791-s001.zip › ijms-1878463-supplementary.pdf]

International Journal of
Molecular Sciences

MDPI

Supplementary data

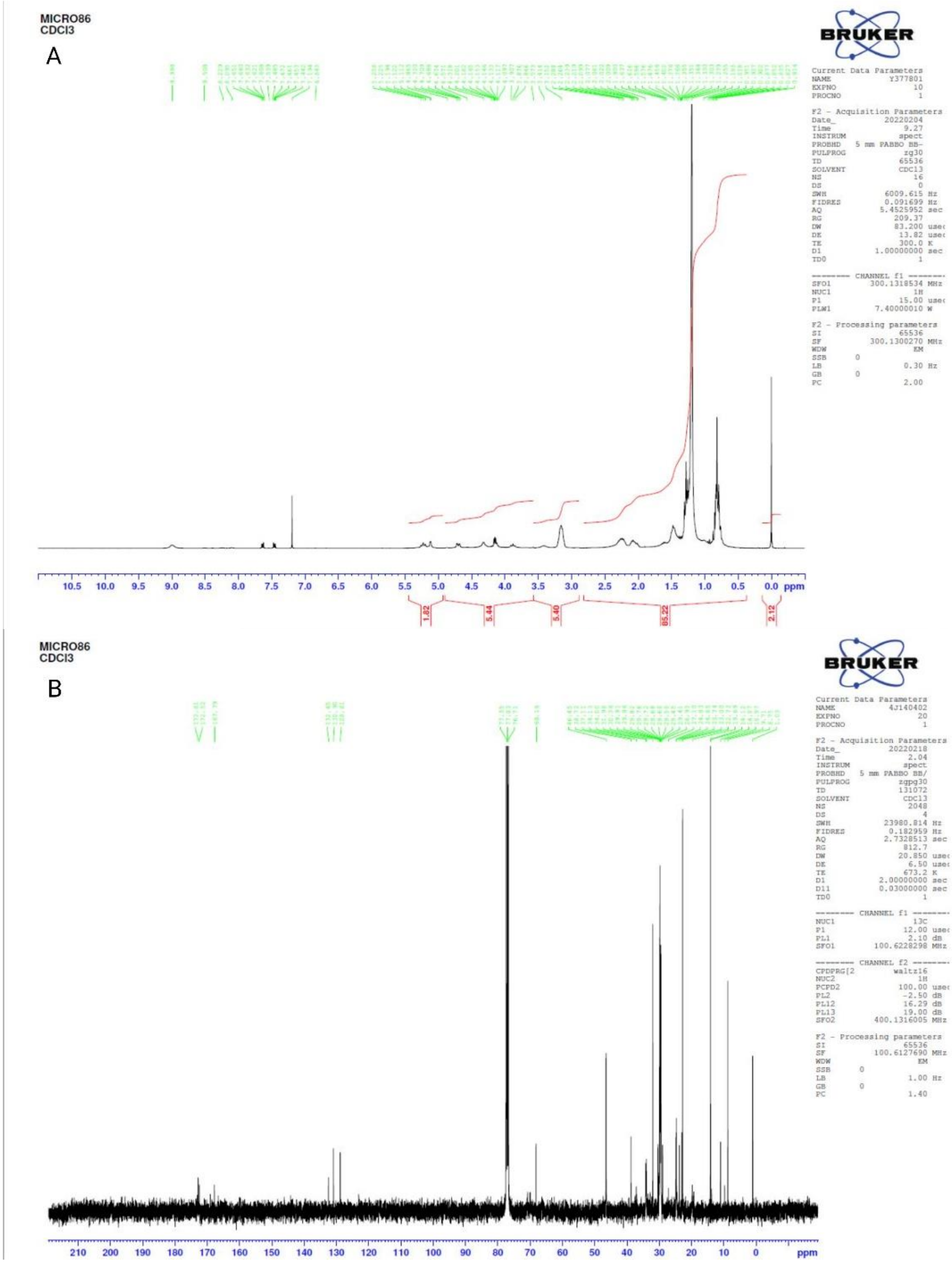

Figure S1. ^{1}H-NMR (A) and ^{13}C-NMR (B) spectra of the amphiphilic anionic CD.

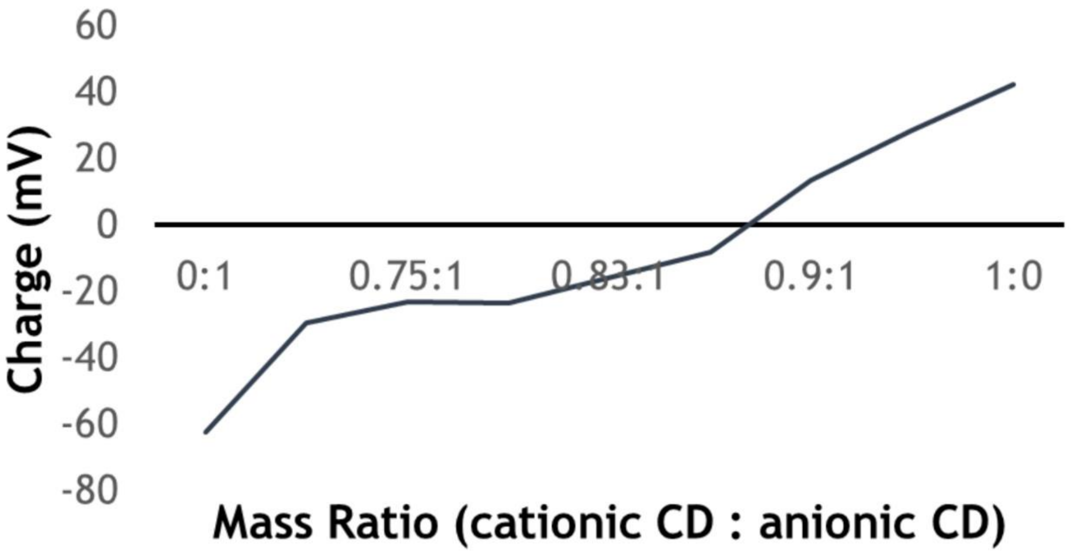

Figure S2. Behaviour of charged CDs in the absence of siRNA. Interaction between anionic and cationic amphiphilic CDs resulted in neutralisation at mass ratio 0.85:1 (cationic CD:anionic CD).

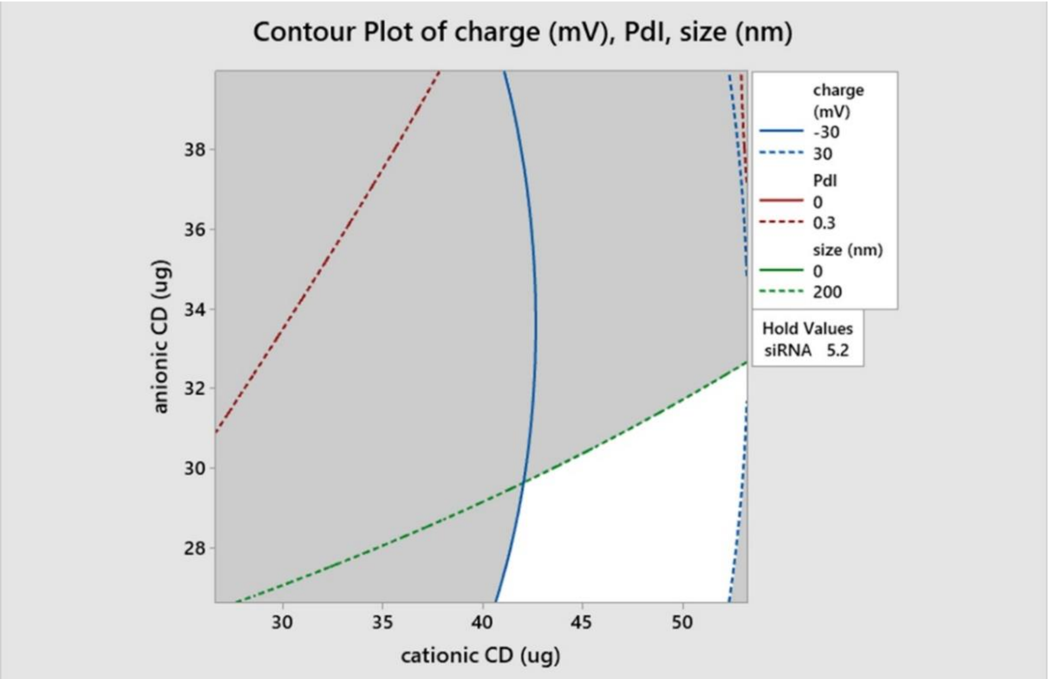

Figure S3. DoE assisted optimisation of mass ratio using Minitab Statistical Software. A 3-factor, 3-level Box Behnken design [49] (Minitab 17) was used. A contour plot with a hold value for siRNA at 5.2 µg (aiming 100 nM siRNA/well) and boundaries for charge between -30 mV to 30 mV, size up to 200 nm and PdI between 0-0.3 were used to determine the optimum mass ratio.

A 3-factor, 3-level Box Behnken design [49,50] (Minitab 17) was utilised to optimize the MR of the siRNA and both CDs. Physicochemical characteristics including size, polydispersity index (PDI) and surface charge (dependent variables), were measured using dynamic light scattering (DLS) to investigate how independent variables (siRNA, amphiphilic cationic CD, and amphiphilic anionic CD quantity) affect the formulation development. Contour plots were used to indicate the optimal MR for the NPs for the in

vitro cell culture studies. In vitro tests were conducted with the two formulations before and after adding the anionic CD.

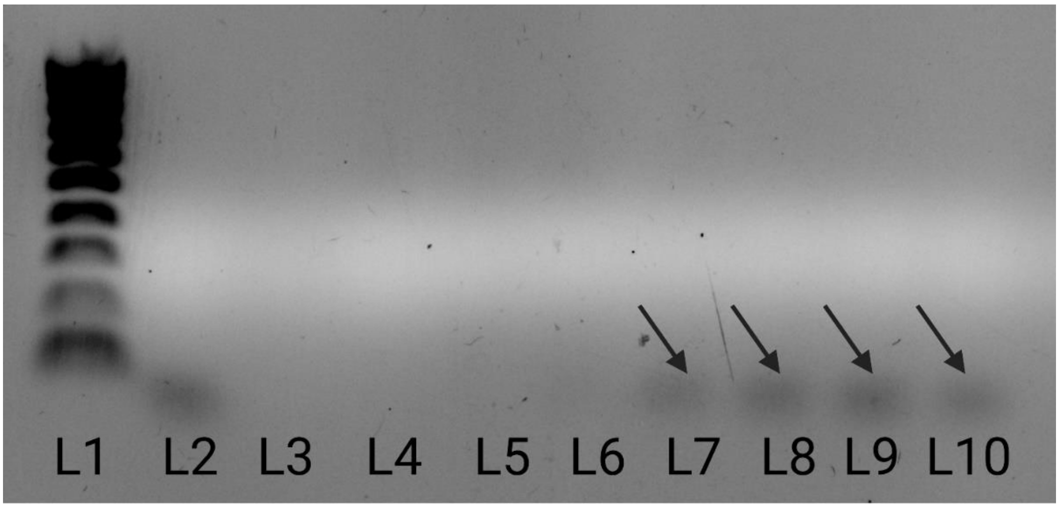

Figure S4. Assessment of higher amphiphilic anionic CD mass ratio (cationic CD:siRNA:anionic CD). A 1 %-Agarose gel was run at 90 V for 60 mins and imaged under UV. It could be seen that at a mass ratio of cationic CD to anionic CD higher than 1.67, the anionic CD displaces the siRNA (circled). L1 100 bp – 1000 bp DNA ladder; L2 siRNA control; L3 NP 10:1 (cationicCD:siRNA); L4 10:1:5; L5 10:1:6; L6 10:1:7.5; L7 10:1:10; L8 10:1:12.5; L9 10:1:15; L10 10:1:17.5.

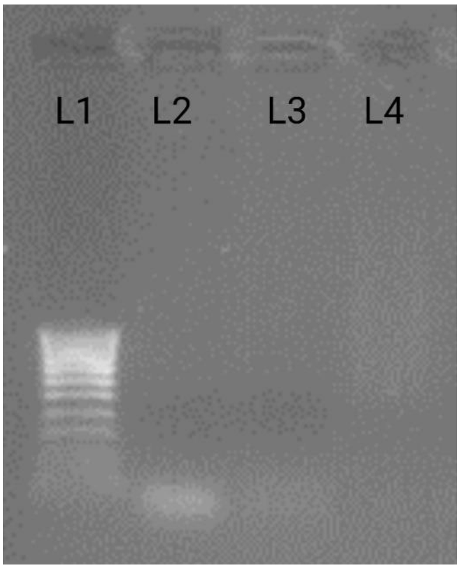

Figure S5. Complexation efficiency using 1%-Agarose gel. L1: 100 bp – 1000 bp DNA ladder, L2: siRNA control, L3: Formulation, L4: Co-formulation

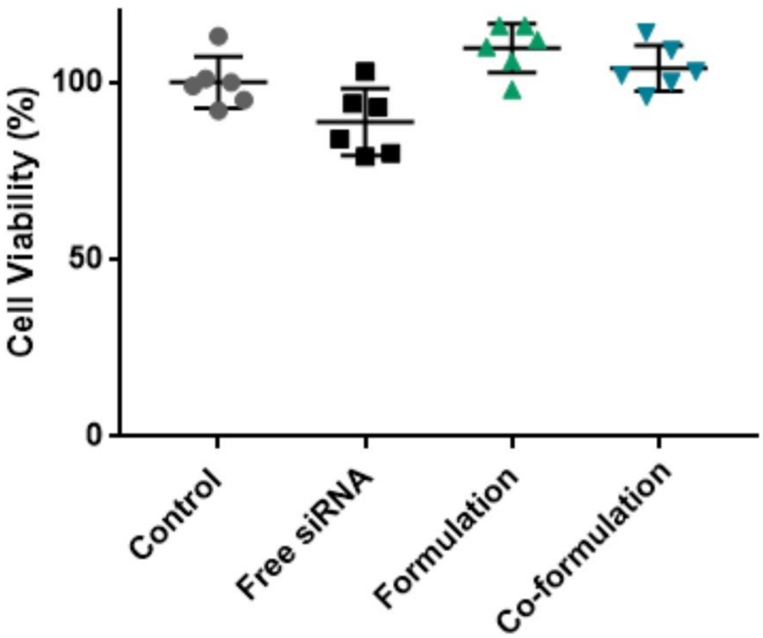

Figure S6. Cytotoxicity assessment. Cell viability (as percentage relative to the control) in HL-60 cells (2.5×10^5/mL) measured 72 h post-transfection using Cell Counting kit 8 (CCK8). Cells were treated with cyclodextrin-nanoparticles at a concentration of 100 nM/well. Both formulations were safe to be used. Mean ± S.E.M of 3 technical replicates performed in triplicate. One-way ANOVA with Dunnett's post hoc test was used. * $p < 0.05$ compared to untreated group. Formulation mass ratio 8.5:1 (cationic CD:siRNA); Co-formulation mass ratio 8.5:1:5.3 (cationic CD:siRNA: anionic CD).
